# Supplementary material for: Comparative Analysis of CXCR5 Circulating DNA Methylation Levels in Autoimmune Rheumatic Diseases
Source: Immun Inflamm Dis. 2025 Jan 21;13(1):e70128. doi: 10.1002/iid3.70128 (PMC11748209; doi:10.1002/iid3.70128)
Supplement: Supplementary file 1 — Supporting information. [file IID3-13-e70128-s001.docx]

**Comparative Analysis of CXCR5 Circulating DNA Methylation Levels**

**in Autoimmune Rheumatic Diseases**

Yiming Shi^1,2,3#^ , Yingying Qin^1,3#^, Yunshen Li^1,2,3#^, Ping Jiang^1,2,3^, Kai Wei^1,2,3^, Jianan Zhao^1,2,3^ , Yu Shan^1,2,3^, Yixin Zheng^1,2,3^, Fuyu Zhao^1,2,3^, Mi Zhou^1,3^, Li Li^3^, Yu Shen^3^, Xinliang Lv^4^, Yuejuan Zheng^5,6^, Shicheng Guo^1^, Qin Ding^1,3*^, Cen Chang^1,3*^, Dongyi He^1,2,3*^

1, Department of Rheumatology, Shanghai Guanghua Hospital of Integrative Medicine, Shanghai University of Traditional Chinese Medicine, Shanghai, China.

2, Guanghua Clinical Medical College, Shanghai University of Traditional Chinese Medicine, Shanghai, China

3, Institute of Arthritis Research in Integrative Medicine, Shanghai Academy of Traditional Chinese Medicine, Shanghai, China.

4,Traditional Chinese Medicine Hospital of Inner Mongolia Autonomous Region, Hohhot, Inner Mongolia Autonomous Region, 010020, P.R. China.

5,The Research Center for Traditional Chinese Medicine, Shanghai Institute of Infectious Diseases and Biosecurity, Shanghai University of Traditional Chinese Medicine, Shanghai, 201203, China

6,Center for Traditional Chinese Medicine and Immunology Research, School of Basic Medical Sciences, Shanghai University of Traditional Chinese Medicine, Shanghai 201203, China

# These authors contributed equally to this work

*Correspondence:

Dongyi He, M.D., Ph.D.

Department of Rheumatology

Shanghai Guanghua Hospital, Shanghai University of Traditional Chinese Medicine

Shanghai, China

Email: dongyihe@medmail.com.cn

Cen Chang, Ph.D.

Department of Rheumatology

Shanghai Guanghua Hospital, Shanghai University of Traditional Chinese Medicine

Shanghai, China

Email: [changcen0928@163.com](mailto:changcen0928@163.com)

Qin Ding

Department of Rheumatology

Shanghai Guanghua Hospital, Shanghai University of Traditional Chinese Medicine

Shanghai, China

Email: dingqin1984@126.com

**Abstract:**

**Objective**: To assess CXC chemokine receptor 5 (CXCR5) circulating DNA methylation differences in autoimmune rheumatic diseases and their relation with clinical features.

**Methods**: Targeted methylation sequencing was performed using peripheral blood from 164 Rheumatoid Arthritis (RA), 30 Systemic Lupus Erythematosus (SLE), 30 Ankylosing Spondylitis (AS), 30 Psoriatic Arthritis (PsA), 24 Sjögren’s Syndrome (SS) patients, and 30 healthy controls (HC).

**Results**: Significant differences in CXCR5 cg19599951 methylation were found between RA and HC, as well as AS and SLE. RA patients exhibited higher methylation than HC and AS (*P* < 0.01) but lower than SLE (*P* < 0.05). SLE patients showed higher methylation compared to HC, AS, and PsA (*P* < 0.001, 0.01, and 0.05, respectively). No significant differences were found in patients with SS compared to other autoimmune diseases and HC. Methylation at cg19599951_103 (r=0.17, *P*<0.05) and cg19599951_209 (r=0.22, *P*<0.01), along with the CC haplotype (r=0.21, *P*<0.01), showed significant positive correlations with erythrocyte sedimentation rate (ESR), while the CT (r=-0.27, *P*<0.001) and TT haplotypes (r=-0.19, *P*<0.05) were negatively correlated. For C-reactive protein (CRP), methylation at cg19599951_103 (r=0.29, *P*<0.001) and cg19599951_209 (r=0.33, *P*<0.0001), and the CC haplotype (r=0.34, *P*<0.0001) were positively correlated, whereas the CT (r=-0.36, *P*<0.0001) and TT (r=-0.30, *P*<0.0001) haplotypes were negatively correlated. Significant negative correlations were observed between the CT haplotype and rheumatoid factor (r=-0.25, *P*<0.01), and anti-citrullinated protein antibody (r=-0.20, *P*<0.05). No significant correlations were found in patients with SLE, AS, and SS. Receiver operating characteristic analysis showed CXCR5 methylation could classify patients with RA vs. those with AS (AUC: 0.624 to 0.967).

**Conclusion**: Differential circulating CXCR5 methylation levels are observed in autoimmune rheumatic diseases which are correlated with inflammatory mediators in RA and may serve as potential biomarkers for RA diagnosis.

**Keywords:** autoimmune rheumatic diseases; DNA methylation; CXCR5; circulating methylation levels; inflammation

**Introduction**

Autoimmune rheumatic diseases (ARDs) are a heterogeneous group of autoimmune disorders that can affect multiple organ systems, particularly joints, muscles, and connective tissues, exhibiting diverse clinical manifestations and often sharing overlapping symptoms, thereby complicating diagnosis. This group includes, but is not limited to, common conditions such as Rheumatoid Arthritis (RA), Systemic Lupus Erythematosus (SLE), Sjögren's Syndrome (SS), and Ankylosing Spondylitis (AS)[1]. The pathogenesis of ARDs is complex and not entirely understood, involving chronic autoimmune inflammation triggered by inappropriate innate and adaptive immune responses against self-tissue[2].

DNA methylation is an epigenetic modification characterized by the covalent attachment of a methyl (-CH3) group to the fifth carbon atom of cytosine bases. This biochemical alteration, while preserving the nucleotide sequence integrity, modulates gene expression patterns by influencing the transcriptional accessibility of genomic regulatory elements[3]. Growing evidence indicates that epigenetic modifications, especially changes in DNA methylation patterns, influence the etiology of ARDs. Furthermore, multiple studies have established a strong correlation between DNA methylation profiles and diverse clinical outcomes and characteristics of ARDs, including disease subtypes and therapy responses[4-6]. Consequently, examining DNA methylation alterations in various ARDs may improve our comprehension of the epigenetic function in ARD pathogenesis.

The CXC chemokine receptor 5 (CXCR5) is an important G protein-coupled receptor (GPCR) family surface protein that regulates the immune system by facilitating the movement and localization of B cells and follicular helper T cells (Tfh), essential for the establishment and upkeep of germinal centers in secondary lymphoid tissues, such as lymph nodes[7]. In normal immune responses, CXCR5 helps guide B cells and Tfh cells to these centers, promoting antibody production and the formation of immune memory. However, in the context of autoimmune diseases, CXCR5-mediated cell localization may lead to abnormal antibody production, attacks on self-tissue, or excessive inflammatory responses, thereby promoting disease development and persistence [8]. Recently, research has shown a close association between CXCR5 and the pathogenesis of SLE, RA, and SS. Patients with RA show increased CXCR5 expression in synovial tissue, yet have fewer CXCR5+ B lymphocytes in peripheral blood [9]. The renal cortex of patients with SLE also shows an increased expression of CXCR5 and its specific ligand CXCL13. Elevated levels of CXCL13 are considered a hallmark of SLE and are associated with disease activity [10-11]. CXCR5 is also identified as a genetic risk factor for SS, with CXCR5+ B cells and T cells accumulating in the target organ (the salivary glands) in SS[12-13]. Previous studies have revealed significant differences in methylation levels at the cg04537602 site of the CXCR5 gene between patients with RA, healthy controls (HC), and patients with osteoarthritis, with significant correlation between methylation levels and inflammation levels in patients with RA [14]. However, it is unclear whether methylation of the CXCR5 varies among different autoimmune rheumatic diseases. Therefore, we included six different populations in this study, namely patients with RA, SLE, AS, Psoriatic Arthritis (PsA), SS, and HC, to investigate and compare the methylation levels of the CXCR5 gene site cg19599951 in peripheral blood. The study explored the relationship between methylation levels and clinical characteristics of these diseases, focusing on inflammatory markers like C-reactive protein (CRP) and erythrocyte sedimentation rate (ESR), to potentially elucidate the role of methylation changes in the pathogenesis of various autoimmune diseases.

**Methods**

**Study Subjects**

The study included 164 patients with RA, 30 with AS, 30 with SLE, 30 with PsA, 24 with SS, and 30 HC (**Table 1**). All participants were recruited from the Precision Medicine Research Cohort at Guanghua Hospital, Shanghai. Patients with RA were categorized into four subgroups according to antibody levels: both rheumatoid factor (RF) and anti-cyclic citrullinated peptide (CCP) antibody positive (RA-DP), double negative (RA-DN), RF negative only (RF-), and CCP negative only (CCP-). Patients with RA fulfilled the American College of Rheumatology (ACR)/European League Against Rheumatism (EULAR) 2010 classification criteria, patients with AS conformed to the modified 1984 New York criteria, patients with SLE were diagnosed with 2019 ACR/EULAR criteria, patients with PsA fulfilled the 2006 International Classification Criteria for Psoriatic Arthritis (CASPAR), and the 2016 ACR/EULAR guidelines were used to stratify individuals with SS. HC were healthy individuals screened at Guanghua Hospital. Exclusion criteria included individuals under 18 years of age, more than one ARD, acute/chronic infections, cancer, and pregnancy or planned pregnancy. All participants provided peripheral blood samples, which were then preserved at -80℃. Additionally, demographic and clinical data were gathered simultaneously.

**Targeted Methylation**

The cg19599951 site is situated within the promoter region of CXCR5, spanning chromosome 11 (chr11): 118883469 to chr11: 118883705, with a length of 237 bp. We extracted the genomic DNA from each peripheral blood sample using a DNA Extraction Kit (TIANGEN Biotech, Beijing, China). The quality of the extracted genomic DNA was assessed using Nanodrop 2000, with concentration requirements of ≥ 20 ng/µL, alongside a total amount ≥ 400 ng. Sample purity was set at OD260/280 ratios of 1.7 to 1.9, and OD260/230 ratios ≥ 2.0. The target region's methylation-specific primers were designed utilizing the Methylation FastTarget V4.1 program. Primer sequences were as follows: Forward Primer: 5' GAAAATGAAGGTTTGGAGGTGGT 3', Reverse Primer: 5' ACTAATCAAACATTTCAACT 3'. The EZ DNA Methylation-Gold Kit (ZYMO RESEARCH, CA, USA) was used for the bisulfite conversion of unmethylated cytosines (C) to uracils (U) in genomic DNA. Bisulfite treatment was followed by two rounds of Polymerase Chain Reaction (PCR). Initially, specific primers and the HotStarTaq polymerase kit (TAKARA, Tokyo, Japan) were used to amplify the target regions. An indexing PCR using Herculase® II Fusion DNA Polymerase (Agilent Technologies, CA, USA) and primers matching the Illumina HiSeq platform was performed in the second PCR stage. This was done in order to pool and amplify all target region amplification products using primers with index sequences adaptable to the Illumina HiSeq platform. PCR reaction conditions are detailed in **Supplementary Materials 2**. First, the PCR products were separated using agarose gel electrophoresis to ensure the acquisition of pure target DNA fragments. Subsequently, gel purification was performed on these separated fragments to remove potential impurities, increasing the purity of the samples. The purified DNA samples underwent 2×150 bp paired-end sequencing on the Illumina HiSeq platform. This method generates high-quality FastQ data, providing a foundation for precise subsequent data analysis.

**Data Analysis**

Data were processed and visualized using IBM SPSS Statistics 27, GraphPad Prism 9, and Sangerbox. Inter-group variations were assessed using the Kruskal-Wallis statistical method. Furthermore, Spearman's rank correlation coefficients and visualization via heatmaps were used to analyze the clinical relevance of methylation levels. Statistical significance was set at *P*-value < 0.05.

**Results**

1. **Differences in CXCR5 Circulating Methylation Levels Among Patients with ARDs**

The analysis of methylation levels at the CXCR5 cg19599951 locus across the different groups indicated median methylation levels of 0.817 (interquartile range, IQR: 0.781-0.841) in HC, 0.839 (IQR: 0.814-0.868) in RA, 0.811 (IQR: 0.788-0.844) in AS, 0.864 (IQR: 0.811-0.900) in SLE, 0.834 (IQR: 0.802-0.850) in PsA, and 0.816 (IQR: 0.784-0.883) in SS. Significant differences were observed in CXCR5 cg19599951 methylation levels between patients with RA and HC, and patients with AS and SLE. Patients with RA exhibited significantly higher methylation compared to HC and patients with AS (*P*=0.0071 and 0.0076, respectively), and significantly lower methylation compared to patients with SLE (*P*=0.0497). Patients with SLE displayed higher methylation levels than HC, patients with AS and PsA (*P*=0.0003, 0.0019, and 0.0249, respectively). Patients with SS did not show significant differences in methylation levels compared with patients of other autoimmune diseases and HC (**Figure 1**).

1. **Differences in CXCR5 Circulating Methylation Levels at Different Sites in Patients with ARDs**

In this study, our candidate CpG sites were cg19599951_103 and cg19599951_209. Methylation levels at site cg19599951_103 showed significant differences between patients with RA and HC (*P*=0.0435), and between patients with SLE and HC (*P*=0.0035). Additionally, the methylation level at this site in patients with SLE showed significant differences when compared with patients with AS and PsA (*P*=0.0053 and 0.012, respectively) (**Figure 2A**). Significant differences were observed at site cg19599951_209 between patients with RA and HC (*P*=0.004), AS(*P*=0.0031), between patients with SLE and HC (*P<*0.0001), as well as patients with AS (*P<*0.0001), PsA (*P*=0.0115) and SS (*P*=0.0115) (**Figure 2B**).

1. **Methylation Haplotype Analysis**

Four methylation haplotypes were identified from the methylation sequencing data, including CC, CT, TC, and TT. Analysis of these haplotypes across groups showed that the proportion of the CC haplotype was higher in patients with RA compared to HC, and patients with AS and PsA(*P* = 0.0025, 0.0094, and 0.0217, respectively); Similarly, in patients with SLE, the proportion of the CC haplotype was higher compared to HC, and patients with AS, PsA and SS (*P*=0.0005, 0.0016, 0.0034, and 0.0231, respectively) (**Figure 3A**). The proportion of the CT haplotype in patients with RA was significantly lower compared to HC, and patients with AS and PsA (*P*=0.0009, 0.0034, and 0.0069, respectively), and the proportion of the CT haplotype in patients with SLE was significantly lower compared to HC, and patients with AS, PsA, and SS (*P*=0.0001, 0.0004, 0.0007, and 0.0075, respectively) (**Figure 3B**). Moreover, the TC haplotype proportion in patients with RA was significantly different compared to patients with SLE, SS, and PsA (*P*=0.0382, 0.0268, and <0.0001, respectively), and the TC haplotype proportion in patients with AS was significantly different compared to patients with SLE, SS, and PsA (*P*=0.0097, 0.0069, and <0.0001, respectively) (**Figure 3C**). On the other hand, the TT haplotype proportion in patients with RA was significantly different compared to patients with AS and SLE (*P*=0.0176 and 0.0035, respectively), and the TT haplotype proportion in patients with AS was significantly different compared to patients with PsA, SS and SLE (*P*=0.0136, 0.0495 and <0.0001, respectively). The TT haplotype proportion was significantly lower in patients with SLE compared to HC (*P*=0.0004) (**Figure 3D**).

1. **Analysis of Differences in Methylation Levels Among Various RA Subgroups relative to Patients with AS and SLE**

Further subgroup analyses of methylation levels in patients with RA showed that the mean methylation levels at the cg19599951 locus in patients with RA-DN, RF-, CCP-, and RA-DP differed significantly from that in patients with AS (P=0.0118, 0.0321, 0.0065, and 0.0041, respectively). There were no discernible variations in methylation levels between patients with SLE and RA subgroups(**Figure 4**).

1. **Correlation Analysis of CXCR5 Circulating Methylation Levels with Clinical Features**

Further investigations were conducted to explore the correlations between average methylation levels at CXCR5 cg19599951, individual sites cg19599951_103 and cg19599951_209, and methylation haplotypes with clinical indices in patients with RA. The average methylation level of CXCR5 was significantly positively correlated with CRP (r=0.34, *P*<0.0001) and ESR (r=0.22, *P*<0.01) (**Figure 5A-B**). Methylation levels at cg19599951_103 (r=0.17, *P*<0.05) and cg19599951_209 (r=0.22, *P*<0.01), as well as the CC haplotype (r=0.21, *P*<0.01), were also significantly positively correlated with ESR. The CT (r=-0.27, *P*<0.001) and TT (r=-0.19, *P*<0.05) haplotypes were significantly negatively correlated with ESR. Methylation levels at cg19599951_103 (r=0.29, P<0.001) and cg19599951_209 (r=0.33, P<0.0001), together with the CC haplotype (r=0.34, P<0.0001), exhibited positive correlations with CRP. The CT (r=-0.36, *P*<0.0001) and TT (r=-0.30, *P*<0.0001) haplotypes were significantly negatively correlated with CRP. Significant correlation was not observed in both 28-joint Disease Activity Score-ESR (DAS28-ESR) and DAS28-CRP with methylation levels (**Figure 6**). No significant correlation was observed after analyzing the average methylation levels at cg19599951, individual sites, and methylation haplotypes with clinical indices in patients with SLE (**Figure S1**), AS (**Figure S2**), and SS (**Figure S3**). However, in patients with PsA, the average methylation level of CXCR5 (r=0.40, *P*<0.05), methylation level at the cg19599951_209 site (r=0.37, *P*<0.05), and the CC haplotype (r=0.40, *P*<0.05) showed a significant positive association with CRP (**Figure S4**).

1. **Receiver operating characteristic (ROC) Analysis**

ROC analysis was performed using individual CXCR5 circulating methylation levels to classify RA and AS, RA-DN and AS, RA-DP and AS, RF- and AS, CCP- and AS, and RA and HC. The area under the curve (AUC) values for the respective groups were 0.662, 0.631, 0.664, 0.624, 0.967, and 0.658. Multivariable logistic regression incorporating age, sex, ESR, CRP, and CXCR5 circulating methylation levels was used to classify patients with RA and AS, and patients with RA-DN and AS, resulting in AUC values of 0.934 and 0.918, respectively (**Figure 7**).

**Discussion**

This study examined the alterations in CXCR5 DNA methylation in peripheral blood of HC and patients with RA, AS, SLE, PsA, and SS. We discovered notable variations in CXCR5 DNA methylation levels across patients with RA and SLE compared with HC, indicating that CXCR5 methylation status may contribute to the pathophysiology of these autoimmune disorders. Furthermore, in line with our earlier results, the overall methylation levels in patients with RA were considerably higher than those in HC. We also noted differences in CXCR5 circulating methylation levels among different ARDs, with increased levels in RA and SLE relative to AS and PsA. This contrast between the high methylation levels in RA and SLE and the relatively low levels in AS and PsA may reflect differences in the pathological mechanisms of these diseases. SLE can impact several organs and systems, including the musculoskeletal, respiratory, and hematologic systems, along with skin and kidneys. It is typically associated with anti-nuclear antibodies and a strong type I interferon response [15-16]. Research indicates that CXCR5 is pivotal in the advancement of lupus by modulating the movement of B cells and double-negative (DN) T cells, thereby promoting germinal center (GC) development and directing pathogenic DN T cells toward lymphoid organs and kidneys[17]. RA is characterized by chronic inflammation and progressive bone destruction[18], with RF and CCP serving as hallmark autoantibodies and diagnostic markers for RA[19]. AS is a common rheumatic disease, predominantly targeting the axial skeleton with chronic inflammation. This condition manifests mainly through spinal involvement and is frequently accompanied by sacroiliitis, uveitis, and enthesitis[20]. AS and PsA belong to the category of spondyloarthritis, conditions in which T lymphocytes, particularly the T helper cell 17 (Th17) subset, are pivotal to disease pathogenesis[21]. Furthermore, the human leukocyte antigen (HLA)-B27 is a major genetic component in the pathophysiology of AS and has high diagnostic importance[22]. Unlike RA and SLE, which are typically associated with a more pronounced autoimmune antibody response, the production of autoantibodies is not a core feature of the pathological process in AS. Changes in methylation may influence the type and intensity of immune responses via gene expression regulation[23], potentially mediating differences in immune regulatory mechanisms across different diseases. The significant differences in CXCR5 circulating methylation observed between RA and SLE relative to spondyloarthritis could impact CXCR5 and its associated immune processes, leading to different disease manifestations. Genome-wide association studies have suggested that genetic variations in CXCR5 may be involved in the pathogenesis of SS[12]. However, our results did not show significant differences in CXCR5 circulating methylation levels among patients with SS. The involvement of additional epigenetic mechanisms in CXCR5 control within the context of SS remains unknown.

Further subgroup analysis based on antibody levels revealed significant differences in CXCR5 circulating methylation levels between patients with RA (whether RA-DP, RA-DN, RF- or CCP- group) and patients with AS. This result reinforced the hypothesis that varying CXCR5 levels in RA versus AS might indicate distinct immune response mechanisms. Incorporating age, sex, ESR, CRP, and CXCR5 circulating methylation levels into a multivariable logistic regression model can effectively distinguish patients with RA from AS, as well as RA-DN from AS. This method shows potential in differentiating these diseases for patients with atypical symptoms and seronegative results in clinical settings. However, our results still need further validation in larger samples.

We detected two CpG sites, cg19599951_103 and cg19599951_209, and four haplotypes: CC, CT, TC, and TT. Our analysis revealed significantly elevated methylation levels at both CpG sites and an increased proportion of the CC haplotype among individuals with RA and SLE compared with HC. Significant differences were observed in the methylation levels at the cg19599951_209 site and the proportion of the CC haplotype between patients with RA and AS, as well as between patients with SLE and both AS and PsA , consistent with our overall analysis of methylation levels. The CC haplotype, representing a fully methylated state, exhibited a significant positive link to ESR and CRP, while the TT haplotype, representing an unmethylated state, showed a negative link to ESR and CRP. This further supported the observed correlation between CXCR5 circulatory methylation changes and inflammation levels in RA.

We observed a significant correlation between peripheral blood CXCR5 cg19599951 methylation levels and inflammatory disease markers in RA patients. This finding was consistent with previous observations at the CXCR5 cg04537602 locus. cg04537602 and cg19599951 both reside within the CXCR5 promoter sequence on chr11. Our analysis confirmed the presence of inflammation-related alterations in the methylation status of the CXCR5 promoter region in the peripheral blood of patients with RA. CRP and ESR are commonly used clinical inflammatory markers that reflect changes in the inflammation level of patients and have been utilized to help assess the inflammatory activity in various rheumatic diseases[24-25]. We searched the Pathobiology of Early Arthritis Cohort (PEAC) database (http://peac.hpc.qmul.ac.uk) and found that peripheral blood CXCR5 mRNA levels were significantly negatively correlated with CRP levels in patients with RA. Despite the relatively weak correlations observed between these indicators, the findings indirectly suggested that elevated CXCR5 methylation could lead to reduced gene expression. Nevertheless, the precise mechanisms by which methylation levels directly regulate gene expression remain unknown. It is important to emphasize that our results merely suggest associations and do not establish direct causal relationships. The DAS is a composite index that assesses disease activity in RA by integrating Tender Joint Counts (TJC), Swollen Joint Counts (SJC), a visual analog scale for patient-reported pain, and CRP or ESR. Although we explored the potential relationship between CXCR5 cg19599951 methylation levels and the DAS28-CRP and DAS28-ESR in patients with RA, our analysis did not reveal any significant associations. Earlier research has revealed links between interferon gene methylation changes in lupus patients and clinical manifestations, including disease severity, kidney complications, and autoantibody production[26]. Our study analyzed the correlation between CXCR5 methylation levels in the peripheral blood of patients with SLE and clinical indicators including ESR, CRP, anti-double stranded DNA (anti-dsDNA) antibody, complement components 3 (C3) and C4, and 24-hour urinary total protein. However, our results did not find a significant correlation between CXCR5 circulating methylation levels and disease activity markers. Clinical correlation analysis between methylation levels of AS and SS was also performed, revealing no direct association. Considering factors such as the number of patients and the specificity of cell types [27], future studies could attempt to increase the sample size, select specific cell types, and longitudinally track the relationship between methylation levels and changes in disease activity.

In summary, our findings revealed significant differences in CXCR5 circulating methylation levels between patients with RA and those with SLE relative to healthy individuals, and notable differences between patients with RA and those with AS and SLE. CXCR5 methylation levels were able to distinctly differentiate patients with CCP-negative RA from those with AS. Additionally, CXCR5 methylation levels in patients with RA were significantly correlated with inflammatory markers. The results further substantiated that alterations in CXCR5 methylation have a role in the pathogenesis of autoimmune illnesses and could potentially serve as markers reflecting the inflammatory levels in RA, assisting disease diagnosis. The underlying mechanisms behind these differential methylation patterns warrant further investigation, especially in terms of how CXCR5 methylation specifically influences immune function and disease progression. Subsequent studies may also explore the methylation status of additional inflammation signaling pathways related to ARDs, to clarify the epigenetic regulatory landscape shaping these pathologies.

**Declarations:**

**Funding:** This research was supported by the National Natural Science Funds of China (82074234, 82073901), the State Administration of Traditional Chinese Medicine, the Shanghai Municipal Health Commission, the East China Region-based Chinese and Western Medicine Joint Disease Specialist Alliance, the National Key Research and Development Program of China (2021YFE0200900), the Shanghai Municipal Health Commission (202340274), the project on traditional therapies and preparations for arthropathy (RCJD2021B04), and the Shanghai Sailing Program (23YF1436300).

**Competing interests:** All authors have actively contributed to the manuscript and provided informed consent for its publication.

**Consent for publication:** All authors claim paper is original and not submitted or published anywhere.

**Authors' contributions:** YS, YQ, and YL collected samples and data and drafted the original manuscript. PJ, KW, ZJ, YS, YZ, FZ, and MZ contributed to sample and data collection, as well as review and editing. LL,YS,YZ and XL for review and editing. SG for conceptualization and review. QD and CC for revising and reviewing the manuscript. DH for the funding acquisition, revision, and manuscript review. The final version was reviewed and approved by all authors.

**Ethics statement:** This study received approval from the Ethics Committee of Guanghua Hospital of Integrated Traditional Chinese and Western Medicine (2020-K-06-01).

**Data access statement:** Data can be accessed by contacting the authors.

**Acknowledgements:** Not applicable.

**Reference**

[1]Barturen G, Beretta L, Cervera R, Van Vollenhoven R, Alarcón-Riquelme ME. Moving towards a molecular taxonomy of autoimmune rheumatic diseases. Nat Rev Rheumatol. 2018 Jan 24;14(2):75-93.

[2]Gerasimova EV, Popkova TV, Gerasimova DA, Kirichenko TV. Macrophage Dysfunction in Autoimmune Rheumatic Diseases and Atherosclerosis. Int J Mol Sci. 2022 Apr 19;23(9):4513.

[3]Tirado-Magallanes R, Rebbani K, Lim R, Pradhan S, Benoukraf T. Whole genome DNA methylation: beyond genes silencing. Oncotarget. 2017 Jan 17;8(3):5629-5637.

[4]Wang Y, Riaz F, Wang W, Pu J, Liang Y, Wu Z, et al. Functional significance of DNA methylation: epigenetic insights into Sjögren's syndrome. Front Immunol. 2024 Mar 6;15:1289492.

[5]Araki Y, Mimura T. Epigenetic Dysregulation in the Pathogenesis of Systemic Lupus Erythematosus. Int J Mol Sci. 2024 Jan 13;25(2):1019.

[6]Ballestar E, Sawalha AH, Lu Q. Clinical value of DNA methylation markers in autoimmune rheumatic diseases. Nat Rev Rheumatol. 2020 Sep;16(9):514-524.

[7]Chu F, Li HS, Liu X, Cao J, Ma W, Ma Y,, et al. CXCR5+CD8+ T cells are a distinct functional subset with an antitumor activity. Leukemia. 2019 Nov;33(11):2640-2653.

[8]Pan Z, Zhu T, Liu Y, Zhang N. Role of the CXCL13/CXCR5 Axis in Autoimmune Diseases. Front Immunol. 2022 Mar 4;13:850998.

[9]Schmutz C, Hulme A, Burman A, Salmon M, Ashton B, Buckley C, et al. Chemokine receptors in the rheumatoid synovium: upregulation of CXCR5. Arthritis Res Ther. 2005;7(2):R217-29.

[10]Schiffer L, Kümpers P, Davalos-Misslitz AM, Haubitz M, Haller H, Anders HJ, et al. B-cell-attracting chemokine CXCL13 as a marker of disease activity and renal involvement in systemic lupus erythematosus (SLE). Nephrol Dial Transplant. 2009 Dec;24(12):3708-12.

[11] Lee HT, Shiao YM, Wu TH, Chen WS, Hsu YH, Tsai SF, et al. Serum BLC/CXCL13 concentrations and renal expression of CXCL13/CXCR5 in patients with systemic lupus erythematosus and lupus nephritis. J Rheumatol. 2010 Jan;37(1):45-52.

[12] Burbelo PD, Ambatipudi K, Alevizos I. Genome-wide association studies in Sjögren's syndrome: What do the genes tell us about disease pathogenesis? Autoimmun Rev. 2014 Jul;13(7):756-61.

[13] Salomonsson S, Larsson P, Tengnér P, Mellquist E, Hjelmström P, Wahren-Herlenius M. Expression of the B cell-attracting chemokine CXCL13 in the target organ and autoantibody production in ectopic lymphoid tissue in the chronic inflammatory disease Sjögren's syndrome. Scand J Immunol. 2002 Apr;55(4):336-42.

[14]Shi Y, Chang C, Xu L, Jiang P, Wei K, Zhao J, et al. Circulating DNA methylation level of CXCR5 correlates with inflammation in patients with rheumatoid arthritis. Immun Inflamm Dis. 2023 Jun;11(6):e902.

[15]Tsokos GC, Lo MS, Costa Reis P, Sullivan KE. New insights into the immunopathogenesis of systemic lupus erythematosus. Nat Rev Rheumatol. 2016 Nov 22;12(12):716-730.

[16] Eloranta ML, Rönnblom L. Cause and consequences of the activated type I interferon system in SLE. J Mol Med (Berl). 2016 Oct;94(10):1103-1110.

[17]Wiener A, Schippers A, Wagner N, Tacke F, Ostendorf T, Honke N, , et al. CXCR5 is critically involved in progression of lupus through regulation of B cell and double-negative T cell trafficking. Clin Exp Immunol. 2016 Jul;185(1):22-32.

[18]Walsh NC, Crotti TN, Goldring SR, Gravallese EM. Rheumatic diseases: the effects of inflammation on bone. Immunol Rev. 2005 Dec;208:228-51.

[19]Nielen MM, van Schaardenburg D, Reesink HW, van de Stadt RJ, van der Horst-Bruinsma IE, de Koning MH, et al. Specific autoantibodies precede the symptoms of rheumatoid arthritis: a study of serial measurements in blood donors. Arthritis Rheum. 2004 Feb;50(2):380-6.

[20]Sieper J, Poddubnyy D. Axial spondyloarthritis. Lancet. 2017 Jul 1;390(10089):73-84.

[21]Tsukazaki H, Kaito T. The Role of the IL-23/IL-17 Pathway in the Pathogenesis of Spondyloarthritis. Int J Mol Sci. 2020 Sep 3;21(17):6401.

[22]Zhu W, He X, Cheng K, Zhang L, Chen D, Wang X, et al. Ankylosing spondylitis: etiology, pathogenesis, and treatments. Bone Res. 2019 Aug 5;7:22.

[23]Heintzman DR, Fisher EL, Rathmell JC. Microenvironmental influences on T cell immunity in cancer and inflammation. Cell Mol Immunol. 2022 Mar;19(3):316-326.

[24]Vilá LM, Alarcón GS, McGwin G Jr, Bastian HM, Fessler BJ, Reveille JD, et al. Systemic lupus erythematosus in a multiethnic cohort (LUMINA): XXIX. Elevation of erythrocyte sedimentation rate is associated with disease activity and damage accrual. J Rheumatol. 2005 Nov;32(11):2150-5.

[25]Dima A, Opris D, Jurcut C, Baicus C. Is there still a place for erythrocyte sedimentation rate and C-reactive protein in systemic lupus erythematosus? Lupus. 2016 Oct;25(11):1173-9.

[26]Lanata CM, Chung SA, Criswell LA. DNA methylation 101: what is important to know about DNA methylation and its role in SLE risk and disease heterogeneity. Lupus Sci Med. 2018 Jul 25;5(1):e000285.

[27]Rahmani E, Schweiger R, Rhead B, Criswell LA, Barcellos LF, Eskin E, et al. Cell-type-specific resolution epigenetics without the need for cell sorting or single-cell biology. Nat Commun. 2019 Jul 31;10(1):3417.

**Figure**

**Figure 1. Methylation level of cg19599951 among patients with autoimmune rheumatic disease**

The average methylation level of cg19599951 among patients with autoimmune rheumatic disease , including RA , AS, SLE, PsA, SS patients and HC. RA, Rheumatoid Arthritis; AS, Ankylosing Spondylitis; SLE, Systemic Lupus Erythematosus; PsA, Psoriatic Arthritis; SS, Sjögren's Syndrome; HC, Healthy Controls

A

B

**Figure 2. Methylation levels at different CpG sites in patients with autoimmune rheumatic disease**

A. Methylation levels at cg19599951_103 among among RA , AS, SLE, PsA ,SS patients and HC. B. Methylation levels at cg19599951_209 among RA , AS, SLE, PsA, SS patients and HC. RA, Rheumatoid Arthritis; AS, Ankylosing Spondylitis ; SLE, Systemic Lupus Erythematosus; PsA, Psoriatic Arthritis; SS, Sjögren's Syndrome; HC, Healthy Controls.

A

B

C

D

B

C

B

**Figure 3. Proportion of haplotypes among patients with RA , AS, SLE, PsA ,SS and HC**

1. Proportion of haplotype CC among RA , AS, SLE, PsA ,SS and HC groups; B. Proportion of haplotype CT among RA , AS, SLE, PsA ,SS and HC groups; C. Proportion of haplotype TC among RA , AS, SLE, PsA ,SS and HC groups.

D. Proportion of haplotype TT among RA , AS, SLE, PsA ,SS and HC groups. RA, Rheumatoid Arthritis; AS, Ankylosing Spondylitis ; SLE, Systemic Lupus Erythematosus; PsA, Psoriatic Arthritis; SS, Sjögren's Syndrome; HC, Healthy Controls.

**Figure 4. Methylation levels of cg19599951 among different subtypes of patients with RA, AS , and SLE**

RA-DN, Rheumatoid Factor and anti-cyclic citrullinated peptide antibody negative RA patients; RF-, RA patients negative only for Rheumatoid Factor; CCP-, RA patients negative only for anti-cyclic citrullinated peptide antibody; RA-DP, Rheumatoid Factor and anti-cyclic citrullinated peptide antibody positive RA patients; RA, Rheumatoid Arthritis; AS, Ankylosing Spondylitis ; SLE, Systemic Lupus Erythematosus.


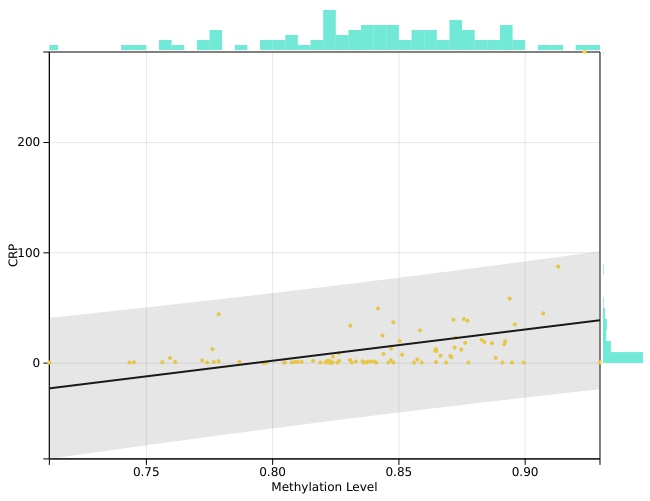


P<0.0001, r=0.34


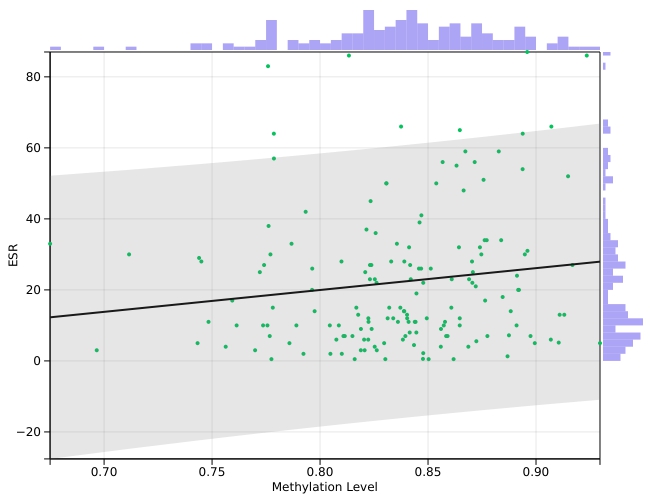


P<0.01, r=0.22

A

B

**Figure 5. The correlation analysis between the methylation level of cg19599951 and inflammatory markers in RA patients**

A. Correlation between methylation level and CRP; B.Correlation between methylation level and ESR.

CRP, C‐reactive protein; ESR, Erythrocyte Sedimentation Rate; RA, Rheumatoid Arthritis.


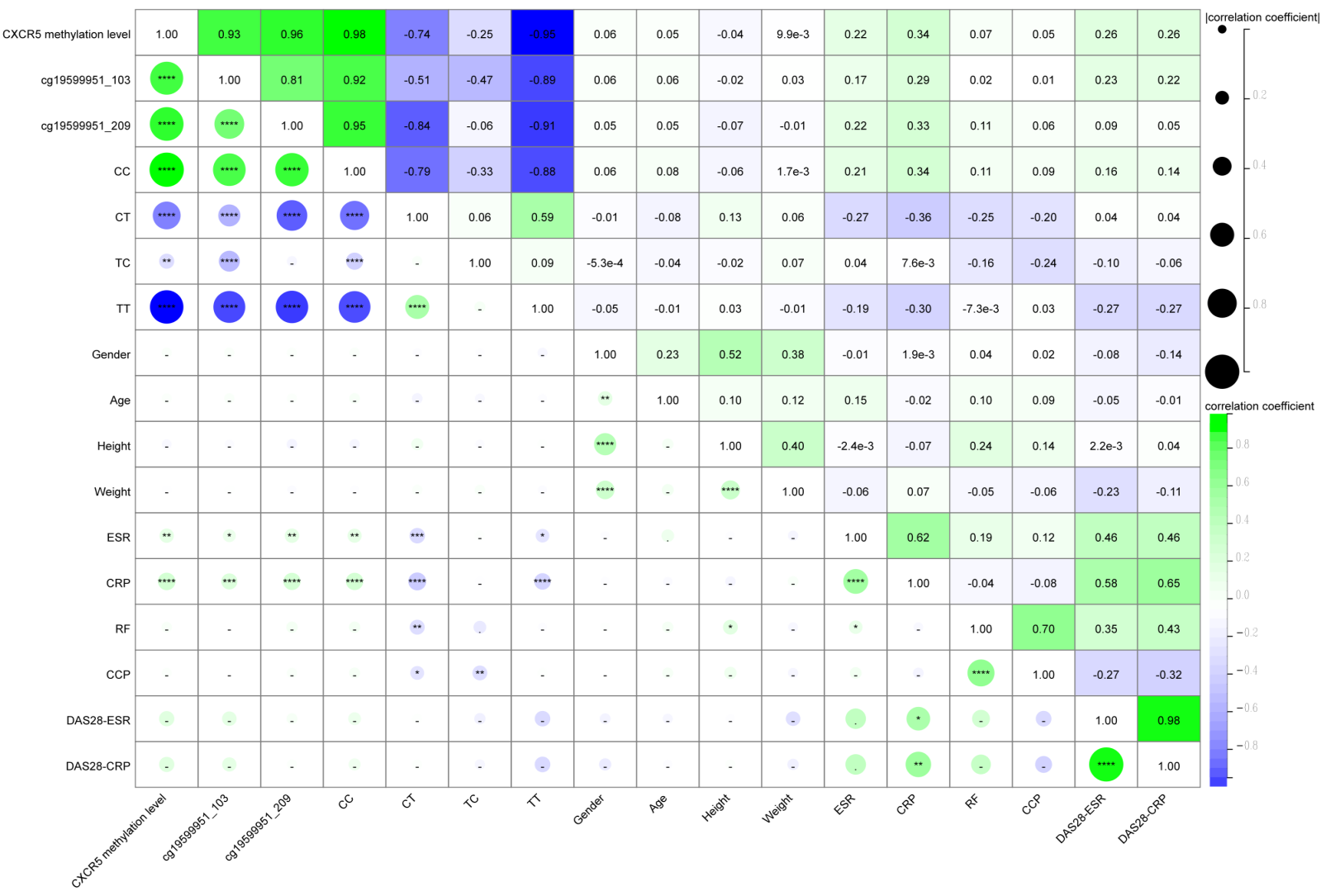


**Figure 6. The correlation analysis between the methylation level of cg19599951 and clinical indicators in RA patients**

RA, Rheumatoid Arthritis; RF, Rheumatoid Factor; CCP, anti-cyclic citrullinated peptide antibody; CRP, C‐reactive protein; ESR, Erythrocyte Sedimentation Rate.


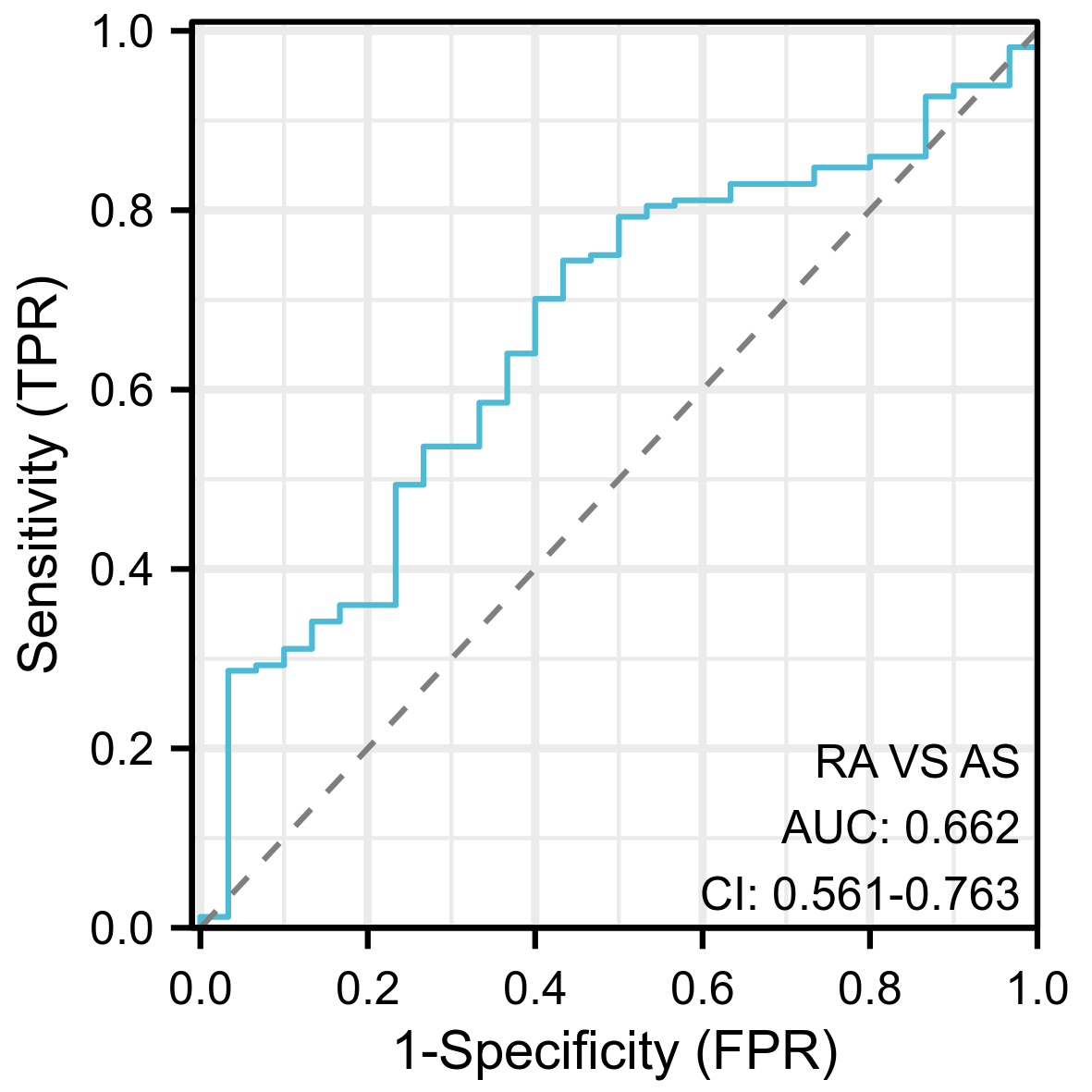

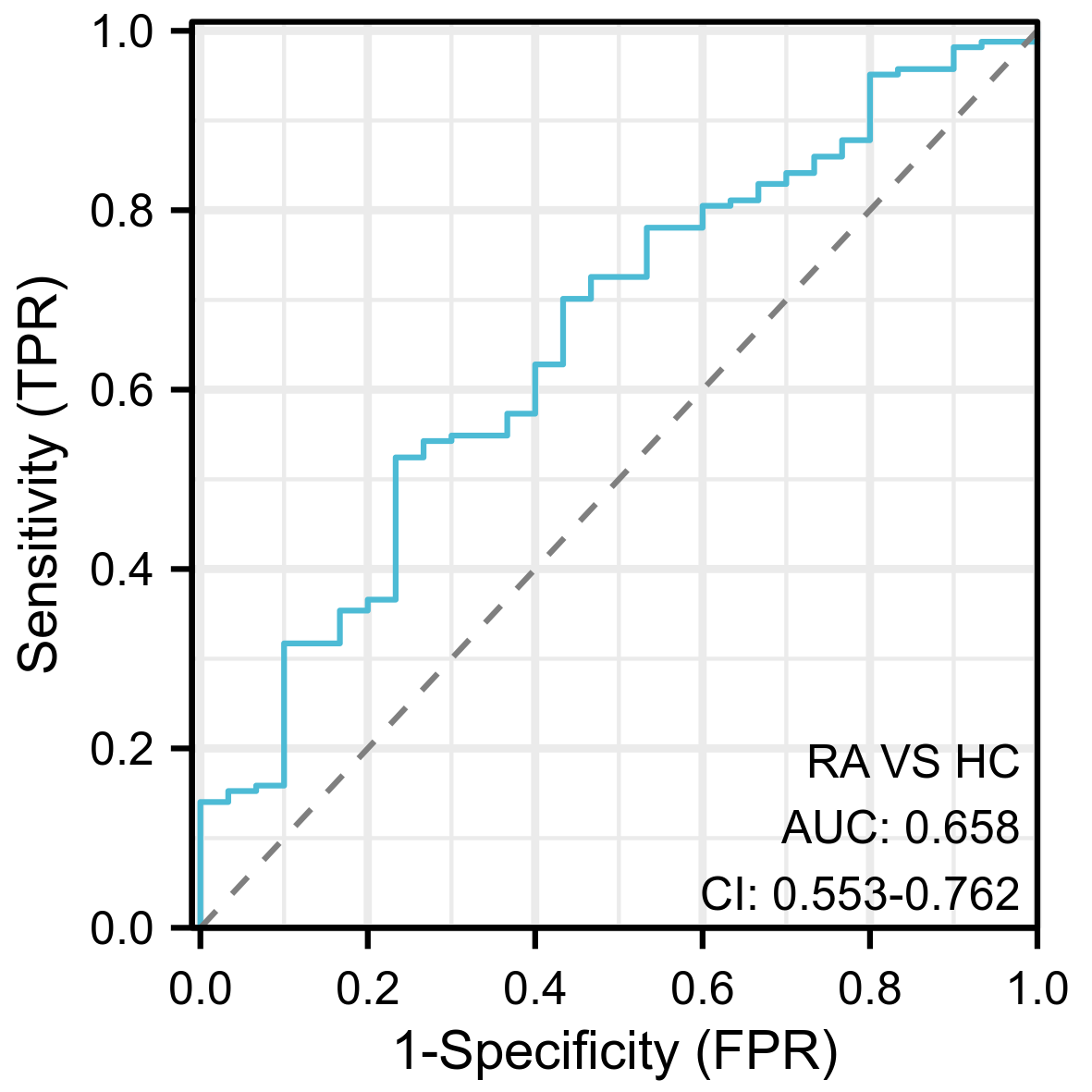

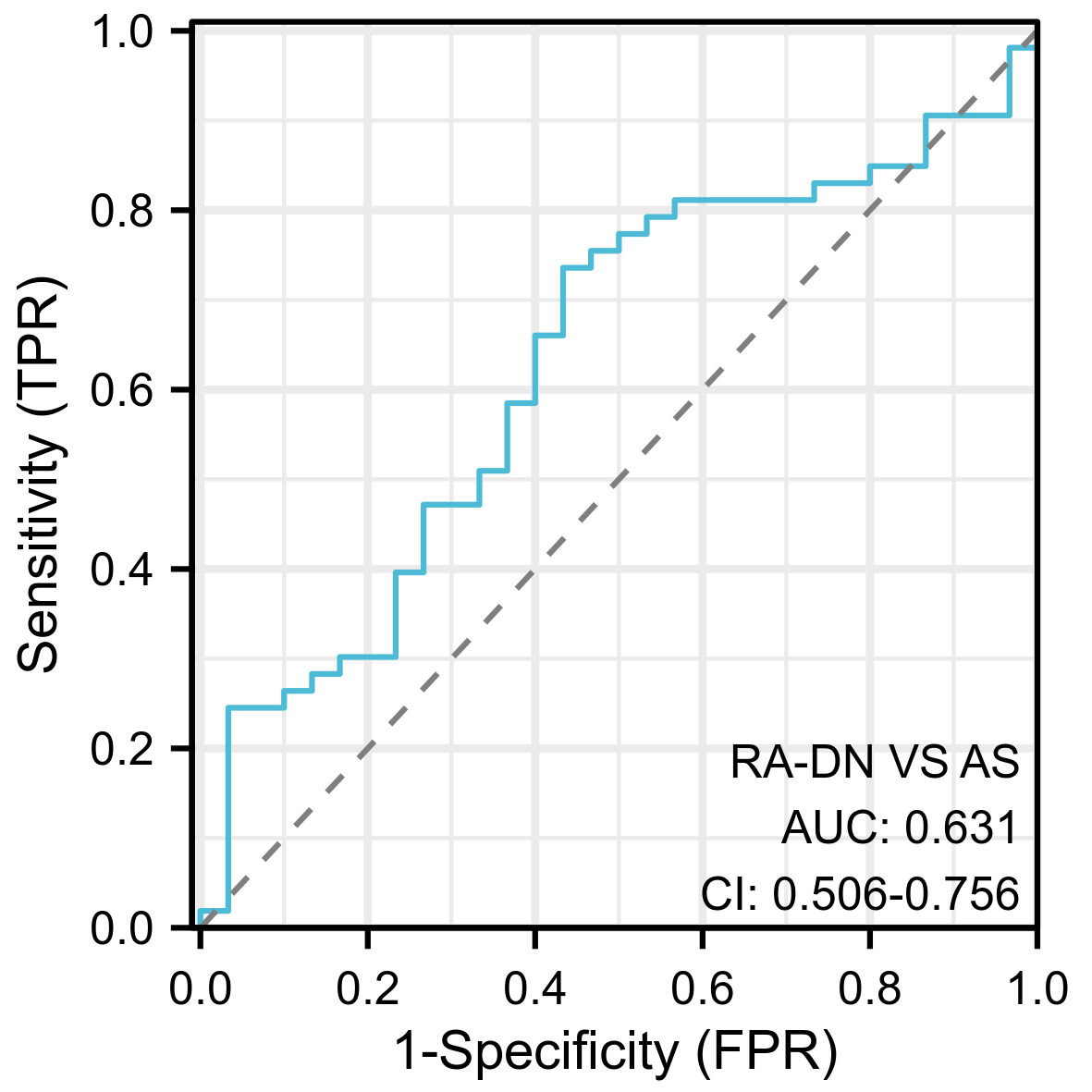

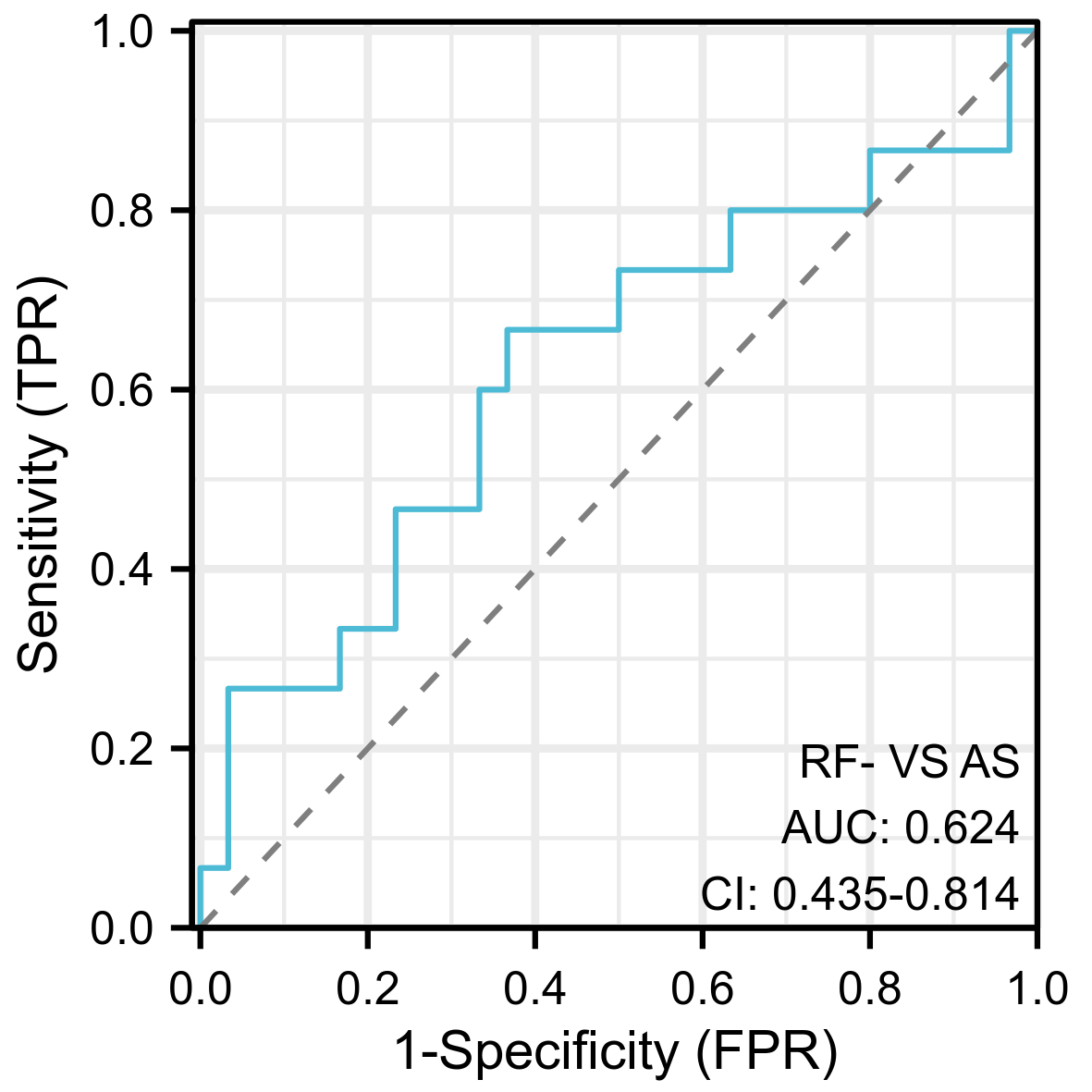

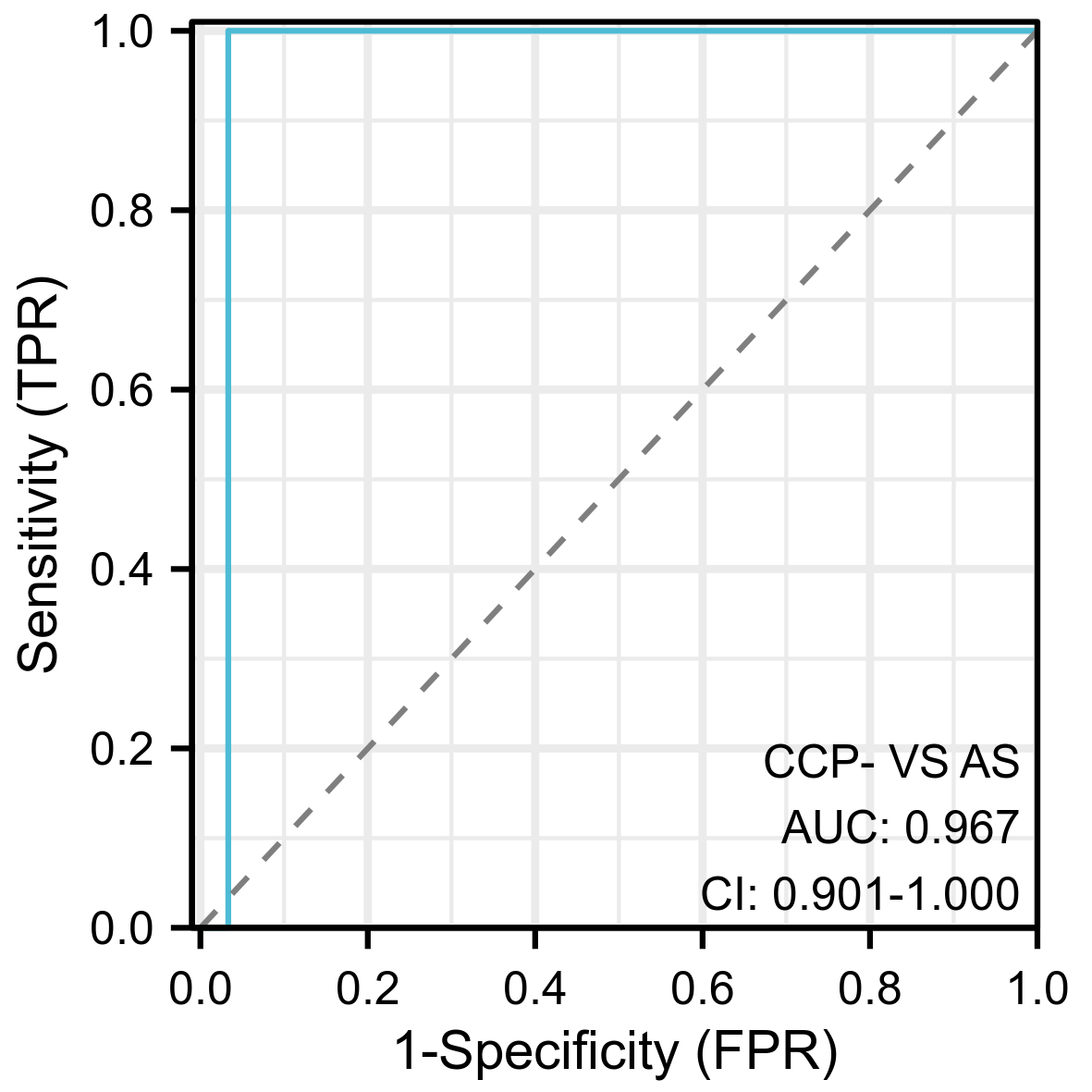

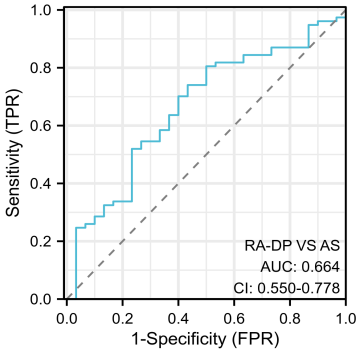

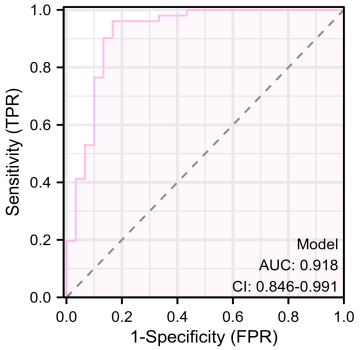

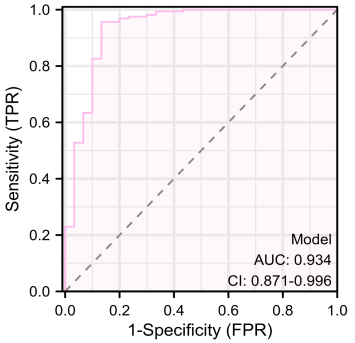


A

B

C

**Figure 7. The ROC curve plot**

A. The ROC curve (left) for distinguishing RA from AS based on the circulating methylation level of CXCR5 alone, and the ROC curve (right) for distinguishing RA from AS based on a multivariate logistic regression incorporating age, gender, ESR, CRP, and circulating methylation levels of CXCR5; B. The ROC curve (left) for distinguishing RA-DN from AS based on the circulating methylation level of CXCR5 alone, and the ROC curve (right) for distinguishing RA-DN from AS based on a multivariate logistic regression incorporating age, gender, ESR, CRP, and circulating methylation levels of CXCR5; C. The ROC curves for distinguishing RA-DP from AS, RF- from AS, CCP- from AS, and HC from AS based on the circulating methylation level of CXCR5 alone.

RA, Rheumatoid arthritis; AS, Ankylosing Spondylitis ; HC, Healthy controls; RA-DN, Rheumatoid Factor and anti-Cyclic Citrullinated Peptide (CCP) antibody negative RA patients; RF-, RA patients negative only for Rheumatoid Factor; CCP-, RA patients negative only for anti-Cyclic Citrullinated Peptide (CCP) antibody; RA-DP, Rheumatoid Factor and anti-Cyclic Citrullinated Peptide (CCP) antibody positive RA patients;

**Table 1 The basic characteristics of participants**

| Items/Group | HC(n=30) | RA(n=164) | AS(n=30) | SLE(n=30) | PsA(n=30) | SS(n=24) |
| --- | --- | --- | --- | --- | --- | --- |
| Gender,n |  |  |  |  |  |  |
| Female | 15 | 139 | 5 | 28 | 6 | 23 |
| Male | 15 | 25 | 25 | 2 | 24 | 1 |
| Height(cm),median (IQR) | 162.50( 158, | 160( 155.5, | 171.5( 167 | 160.5( 156. | 169( 164.5, | 163( 160, 16 |
|  | 170) | 165) | .5, 177) | 8, 163) | 171.3) | 4.5) |
| Weight(kg),median (IQR) | 64(54,69.25 ) | 57(51, 65) | 75(65,85) | 62.5(55.75, 73.5) | 66.5(60,76 .  25) | 54(51,61.5 ) |
| Age(years),median (IQR) | 47.5(41, 52.75) | 60.5(53,69) | 39.5(28.5, 52.75) | 47.5(37.25, 59.25) | 57(46,66) | 60.5(53,65 ) |
| ESR(mm/h),median (IQR) | - | 20( 10,30.5) | 5.50(3.75, 12.25) | 47(15.25,7 5.75) | 7.5(4, 15.5) | 17.5(7,30) |
| CRP(mg/L) ,median (IQR) | - | 1.39(0.5, 11 .16) | 1.31(0.5,6 .46) | 1.04(0.5,8. 51) | 1.31(0.5,3. 8) | 0.54(0.5,3. 3) |
| RF (IU/ml),median (IQR) | - | 20.7(9.19, 108.30) | - | - | - | - |
| CCP(U/ml),median (IQR) | - | 198.2(20, 847.1) | - | - | - | - |
| anti-dsDNA | - | - | - | 9(4.66,62.8 | - | - |
| antibody(IU/ml),median (IQR) |  |  |  | ) |  |  |
| C3(g/L),median (IQR) | - | - | - | 0.76(0.6, 1.  05) | - | - |
| C4(g/L),median (IQR) | - | - | - | 0.19(0.11,0  .30) | - | - |
| 24hUTP(mg/24h),median (IQR) | - | - | - | 278(101.5, 825.3) | - | - |

RF, Rheumatoid Factor; CCP, anti-Cyclic Citrullinated Peptide antibody; ESR, Erythrocyte Sedimentation Rate; CRP, C-reactive protein; anti-dsDNA, anti-double stranded DNA; C3, Complement Component 3; C4, Complement Component 4; 24hUTP, 24-Hour Urinary Total Protein; HC, Healthy controls; RA, Rheumatoid arthritis; AS, Ankylosing Spondylitis ; SLE, Systemic Lupus Erythematosus; PsA, Psoriatic Arthritis; SS, Sjögren's Syndrome.

**Supplementary material 1**


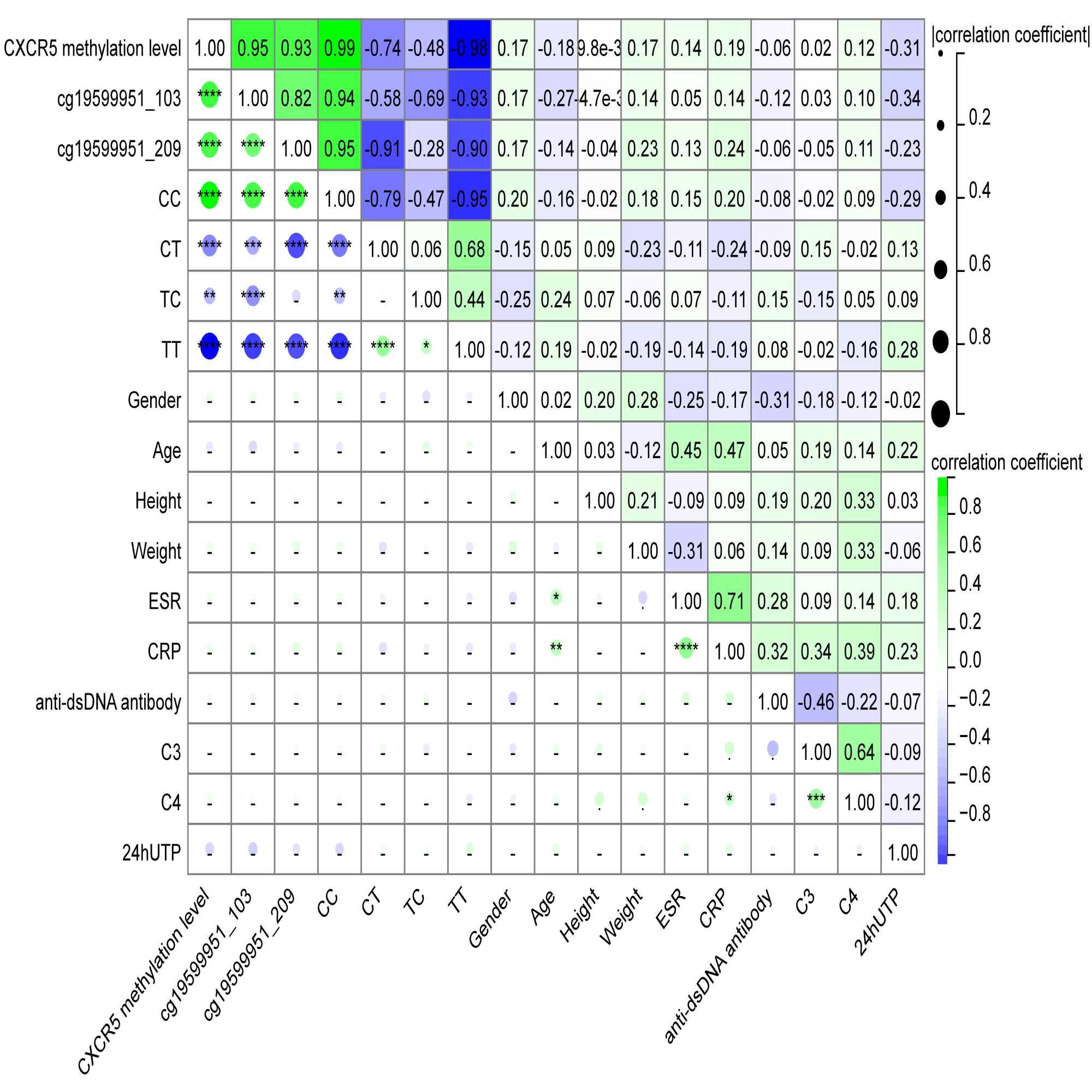


**Figure S1. The correlation analysis between the methylation level of cg19599951 and clinical indicators in SLE patients**

CRP, C‐reactive protein; ESR, Erythrocyte Sedimentation Rate；C3, Complement Component 3; C4, Complement Component 4 ; 24hUTP, 24-Hour Urinary Total Protein; SLE, Systemic Lupus Erythematosus.


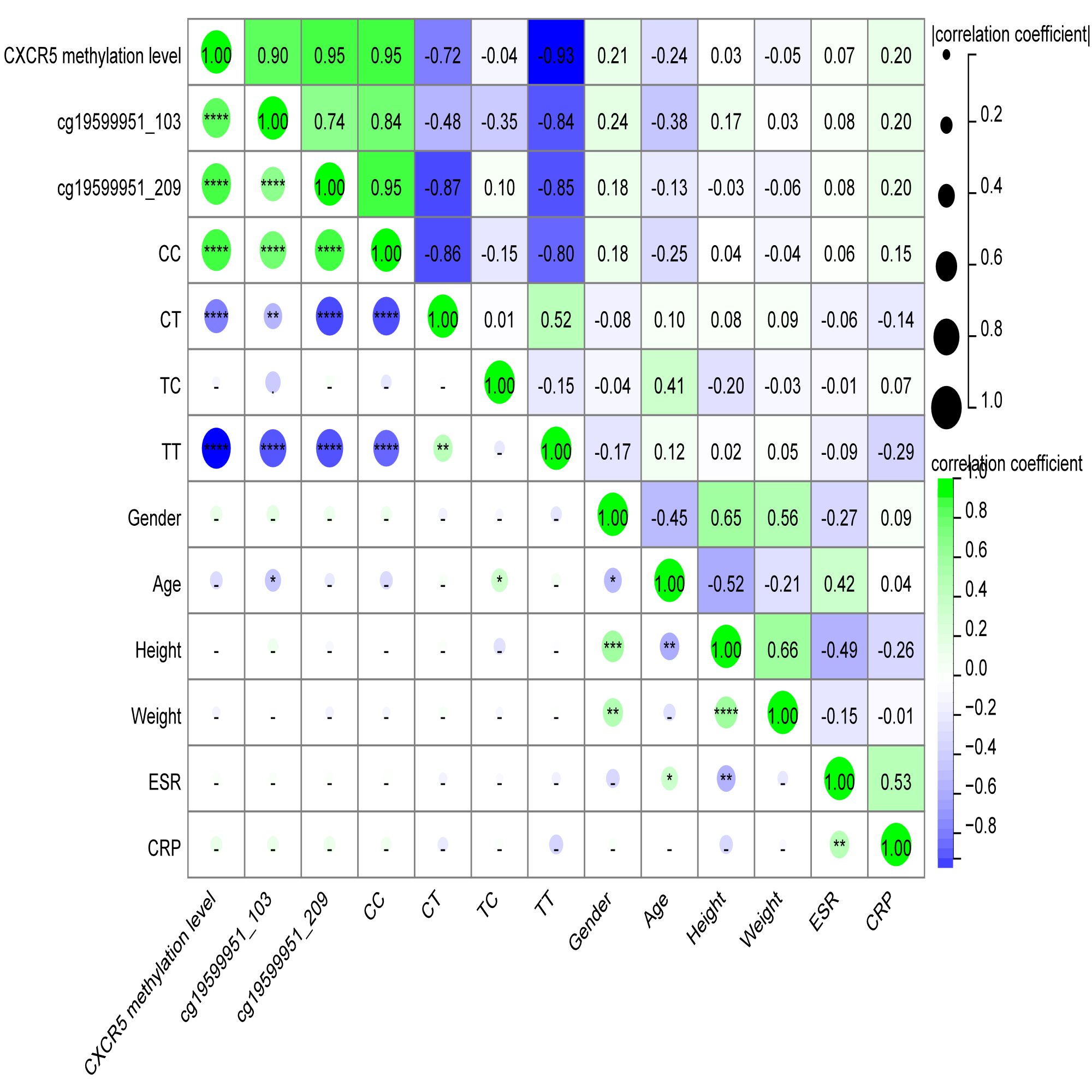


**Figure S2. The correlation analysis between the methylation level of cg19599951 and clinical indicators in AS patients**

AS, Ankylosing Spondylitis; CRP, C‐reactive protein; ESR, Erythrocyte Sedimentation Rate


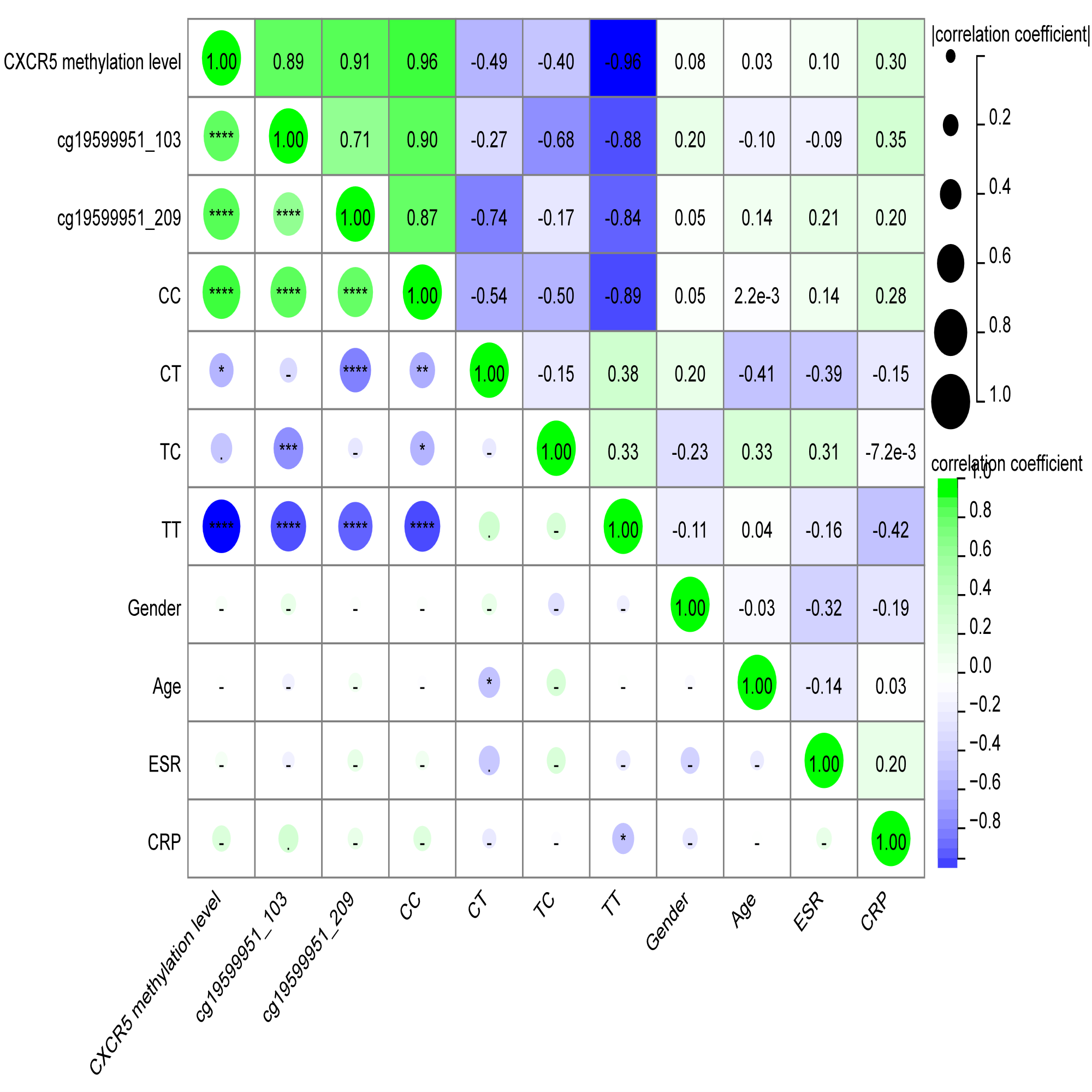


**Figure S3. The correlation analysis between the methylation level of cg19599951 and clinical indicators in SS patients**

CRP, C‐reactive protein; ESR, Erythrocyte Sedimentation Rate; SS, Sjögren's Syndrome.


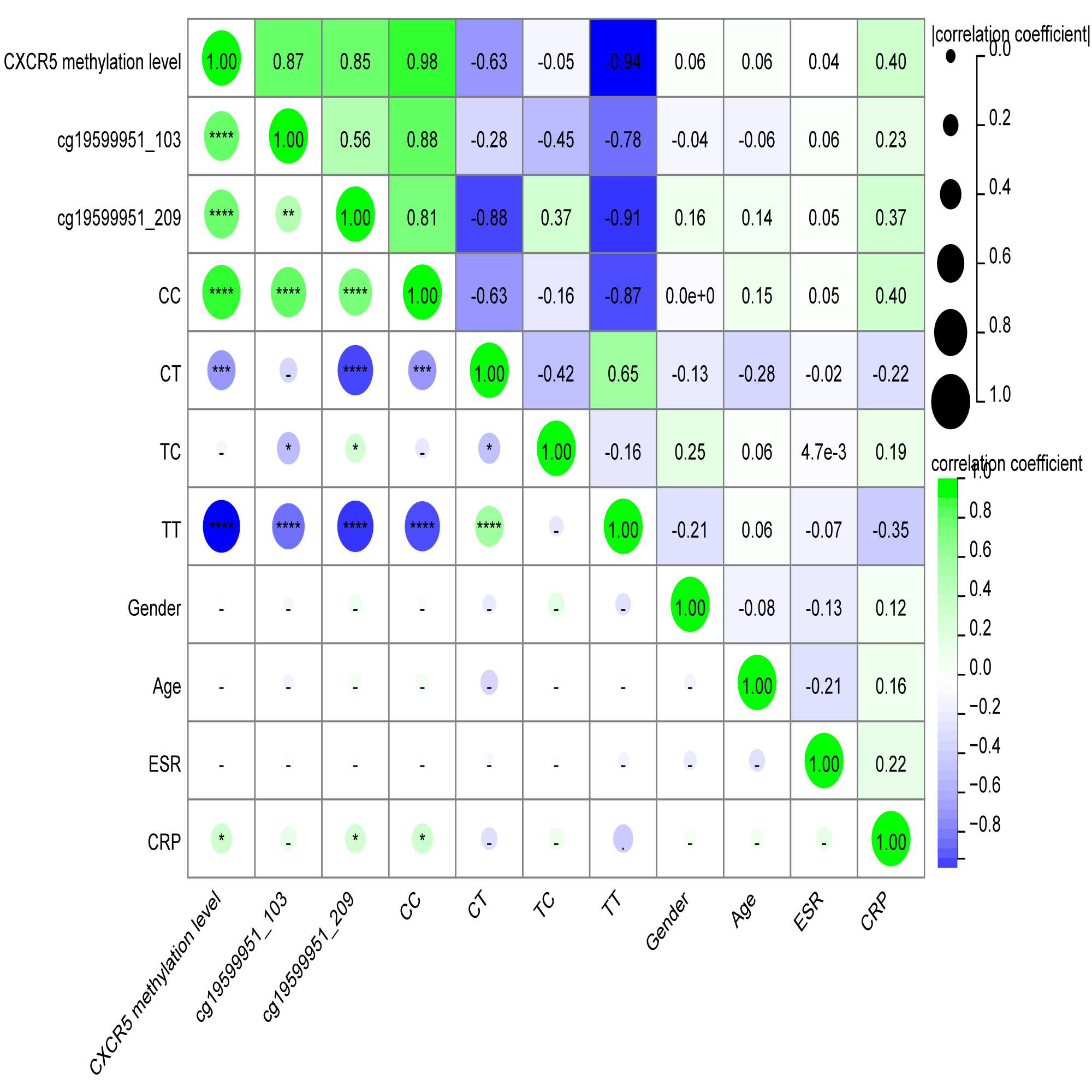


**Figure S4. The correlation analysis between the methylation level of cg19599951 and clinical indicators in PsA patients**

CRP, C‐reactive protein; ESR, Erythrocyte Sedimentation Rate; PsA, Psoriatic Arthritis.

**Supplementary material 2**

**1.PCR amplification of the target fragment**

1. PCR reaction system:

| **Components** | **Volume** |
| --- | --- |
| 10× buffer (TAKARA) | 2μL |
| dNTP (2.5mM) | 2.4μL |
| MgCl2 (25mM) | 1.2μL |
| Gene-specific primers (1μM) | 2μL |
| HotTaq 5U/μL (Takara) | 0.3μL |
| Bisulfite treatment of sample DNA | 1μL |
| ddH2O | 11.1μL |
| **Total volume** | **20μL** |

1. PCR reaction conditions:

|  | **Denaturation** | **Annealing** | **Extension** | **Holding** | **Cycle number** |
| --- | --- | --- | --- | --- | --- |
| 1 Step | 95°C 2min |  |  |  | 1× |
| 2 Step | 95°C 20s | 62°C 40s | 72°C 1min |  | 11× (-0.5°C/cycle) |
| 3 Step | 95°C 20s | 64°C 30s | 72°C 1min |  | 24× |
| 4 Step |  |  | 72°C 1min |  | 1× |
| 5 Step |  |  |  | 4°C | forever |

**2. Index PCR**

1. PCR reaction system:

| **Components** | **Volume（μL）** |
| --- | --- |
| 5× Reaction Buffer | 4μL |
| dNTP (2.5mM) | 2.4 μL |
| NGMPCRF (10μM)* | 0.8μL |
| NGMPCRR  (4 μM)* | 2μL |
| Herculase® II Fusion DNA Polymerase | 0.2μL |
| PCR product after dilution | 2μL |
| ddH2O | 10.6μL |
| **Total volume** | **20** μL |

1. PCR reaction conditions:

|  | **Denaturation** | **Annealing** | **Extension** | **Holding** | **Cycle number** |
| --- | --- | --- | --- | --- | --- |
| 1 Step | 95°C 2min |  |  |  | 1× |
| 2 Step | 95°C 20s | 60°C 40s | 72°C 1min |  | 12 |
| 3 Step |  |  | 72°C 2min |  | 1× |
| 4 Step |  |  |  | 4°C | forever |
